# Lower ribavirin biodisponibility in patients with HIV-HCV coinfection in comparison with HCV monoinfected patients

**DOI:** 10.1186/1471-2334-14-150

**Published:** 2014-03-20

**Authors:** Giorgiana Hatu, François Bailly, Emmanuel Pourcelot, Pierre Pradat, Patrick Miailhes, Marianne Maynard, François Parant, Pierre Chiarello, Jean-Michel Livrozet, Fabien Zoulim, Marie-Claude Gagnieu

**Affiliations:** 1 Department of Hepatology, Hôpital de la Croix-Rousse, Hospices Civils de Lyon, 103 grande rue de la Croix-Rousse, 69004 Lyon, France; 2 Université de Médicine et Pharmacie "Iuliu Hatieganu", Cluj-Napoca, Romania; 3 INSERM U1052, Lyon, France; 4 Université Lyon I, Lyon, France; 5 Pharmacology, Hôpital Edouard Herriot, Hospices Civils de Lyon, Lyon, France; 6 Departement of Infectious Diseases, Hôpital de la Croix-Rousse, Hospices Civils de Lyon, Lyon, France; 7 Department of Immunology, Hôpital Edouard Herriot, Hospices Civils de Lyon, Lyon, France

**Keywords:** Hepatitis C, Human immunodeficiency virus, Ribavirin exposure, Treatment, Coinfection

## Abstract

**Background:**

In HIV infected patients, the impact of ribavirin (RBV) pharmacology on sustained virologic response (SVR) to hepatitis C virus (HCV) treatment has not been fully investigated. The objective of this study was to compare the early RBV plasma exposure between a population of HIV-HCV coinfected patients and an HCV monoinfected group.

**Methods:**

Early RBV plasma exposure (expressed as Area Under the Curve (AUC) from 0 to 4 h) after a 600 mg first dose of RBV was measured in a population of HIV-HCV coinfected patients in comparison with an HCV monoinfected group. Peripheral blood samples were collected before the 600 mg RBV first dose (T0) to ensure no detectable baseline plasma RBV, and then 30 mn, 1, 2 and 4 hours after RBV intake (T0.5, T1, T2 and T4).

**Results:**

Eighty-six patients with chronic hepatitis C entered the study among whom 23 (27%) were HIV-HCV coinfected. Coinfected patients had a significantly lower RBV-AUC _0-4h_ (median: 1469 μg*h/L [range 936–3677]) compared with monoinfected patients (2030 μg*h/L [851–7700]; p = 0.018). This RBV under exposure in coinfected patients persisted after normalization of AUC to RBV dose per kilogram of body weight (182 μg*h/L [110–425] *versus* 271 μg*h/L [82–1091], p = 0.001).

**Conclusions:**

These results suggest that lower early bioavailability of RBV could be one of the reasons for lower SVR in HIV-HCV coinfected patients treated with pegylated interferon/RBV combination therapy. RBV plasma underexposure seems to be associated with the immunological status of the patients with lower AUC_0-4h_ values observed in the more immunosuppressed coinfected patients.

## Background

Worldwide prevalence of hepatitis C virus (HCV) infection in human immunodeficiency virus (HIV) infected patients is about 33%[1-3] and this pathology represents one of the main comorbidities in this population [4,5]. Before the use of protease inhibitors for genotype 1 HCV infection, combination of ribavirin (RBV) and pegylated interferon-α (PEG-IFN) was the standard of care for treatment of chronic HCV patients and it is still the standard for non-1 genotype infection with or without HIV coinfection. All guidelines to adjust RBV doses have been defined in monoinfected patients but are also used in HIV-HCV ones. According to these guidelines and excepted for genotype 2 or 3 infected patients, RBV doses must be adapted to body weight [6]. Studies have shown that high RBV plasma concentrations could improve early virological response rates [7,8]. However, RBV daily dose, even adjusted for body weight is poorly correlated with RBV plasma concentration [9,10] due to a large inter-individual variability in exposure [11-13]. Different explanations have been proposed for this variability: i) the lean body weight would be a better covariate with a linear influence on RBV clearance [14]; ii) RBV exposure depends on creatinine clearance [15]; iii) the RBV pharmacokinetics could be impacted by the pharmacological or pathological context of HIV-HCV coinfection [16]. Loustaud-Ratti et al., showed that RBV plasma exposure after a single dose of 600 mg in HCV monoinfected patients was predictive of sustained virological response (SVR) and proposed a threshold of 1755 μg.h/L for the RBV plasma AUC from 0 to 4 h post-dose to discriminate patients with subsequent SVR from those with non-response [17]. This is currently the only data that can be used to interpret early RBV exposure and determine the optimal dose to initiate treatment. The impact of the pharmacokinetic properties of RBV in HIV patients has not been fully investigated. As for HCV monoinfected patients, we know that a large inter-individual variability in RBV plasma concentrations exists in HIV-HCV coinfected patients and that RBV exposure is associated with both efficacy and anemia [8]. Moreover, the SVR rate is known to be lower in HIV-HCV coinfected than in HCV monoinfected patients. One possible explanation could be that, as for other drugs, HIV infected patients present a lower bioavailability of RBV [18]. The aim of this study was to determine early plasma exposure evaluated by the AUC_0-4h_ after a first dose of RBV in a population of HIV-HCV coinfected patients and to compare the results to those obtained in HCV monoinfected patients.

## Methods

### Patients

Consecutive patients seen by six physicians during a 12 months period for a chronic hepatitis C infection (Edouard Herriot and Croix-Rousse hospitals, Lyon, France) and initiating a PEG-IFN/RBV therapy between February 2009 and February 2010 entered the study. RBV was taken with light breakfast or snack. Plasma AUC determination after a first dose of RBV (600 mg) was obtained before therapy initiation as described by Loustaud-Ratti et al. [17] to evaluate RBV bioavailability. Treatment was then initiated using a weight-based RBV dose after possible adaptation following the AUC results. Patients with low ribavirin exposure (as assessed by AUC result) were usually treated with a 200 mg higher ribavirin dose. Patients were divided into two groups according to their HIV status. To be included, patients had to have detectable HCV RNA, and for HIV-HCV coinfected patients to be on combined antiretroviral therapy (cART) for at least one month prior to RBV initiation. Patients with RBV treatment within the past three months were excluded. All patients gave their informed consent before they entered the study. Following characteristics were recorded at baseline (day of the first RBV dose intake): age, body weight, body mass index (BMI), creatinine level, glomerular filtration rate (GFR, estimated by Cockroft clearance and Modification of the Diet in Renal Disease (MDRD) formula), hemoglobin, aminotransferases, fibrosis stage, HCV genotype, HCV- and HIV RNA quantification, CD4 level and antiretroviral drugs.

### Methods

The plasma AUC was determined for each patient before the antiviral treatment or at the first day. Patients received a single RBV dose of 600 mg (T0) and peripheral blood samples were collected before the administration to ensure undetectable baseline plasma RBV, and then 30 mn, 1, 2 and 4 hours after RBV intake (T0.5, T1, T2 and T4). To avoid RBV exchange between plasma and red blood cells, samples were placed in ice immediately after withdrawal and centrifuged as soon as possible (maximum lag time was 1 h). RBV plasma concentrations were measured using a validated High-Performance Liquid Chromatography-Diode Array Detector method [19]. This method is highly specific, sensible (limit of quantification = 0.05 mg/L), and precise (total imprecision, calculated by measuring the coefficient of variation (CV) of the internal quality control values was less than 10% for concentrations from 0.20 to 5.00 mg/L).

### Pharmacokinetic analysis

RBV AUC was calculated using the Table Curve 2D software (Systat Software Inc). As a large variability of body weight (BW) was observed and that all patients received the same RBV first dose, AUC were also normalized as AUC_0-4h_/dose (in mg/BW in kg). Results of AUC and normalized AUC were analyzed according to the two groups defined by the HIV status.

### Statistical analysis

All quantitative variables were expressed as median and range. Comparisons of data were made using the non-parametric Mann–Whitney test. Statistical analysis was performed using the Statistica® software package (StatSoft Inc). A multivariate logistic regression analysis was conducted to identify factors potentially associated with RBV underexposure (defined by an AUC <1755 μg.h/L). Variables significantly associated with RBV underexposure in univariate analysis and variables suspected to be associated with it, were included in the multivariate model. A p value below 0.05 was considered as statistically significant.

### Ethical considerations

RBV pharmacokinetics monitoring is routinely performed in our unit for patients treated with PEG-IFN/RBV. This monitoring includes measurement of RBV AUC before therapy initiation and assessment of RBV plasma concentration during treatment. RBV monitoring is used to adapt RBV dose and to manage anemia. According to the French legislation (Public Health Code modified by the law no. 2004–806, 9 August 2004 and the Huriet–Sérusclat act 88–1138, 20 December 1988) and since this study was observational, no ethics committee approval was necessary.

## Results

Eighty-six Caucasian patients with chronic hepatitis C entered the study. Among them, 62 were males (72%) and 23 (27%) were HIV-HCV coinfected. Most of them (85%) were infected with HCV genotype 1 or 4. Baseline characteristics are presented in Table 1.

**Table 1:** Main baseline characteristics of HCV monoinfected and HIV-HCV coinfected patients (n = 86)

**Characteristics**	**HCV (n = 63)**	**HIV-HCV (n = 23)**	***p value***
Gender ratio (M/F)	2.0	6.7	0.063
Age (years)	50 *(23–74)*	46 *(35–60)*	0.066
Body weight (kg)	75 *(41–130)*	69 *(52–96)*	**0.032**
*Men body weight (kg)*	*79 (44–130)*	*69 (52–96)*	***0.008***
Body mass index (BMI)	25.7 *(16.4-43.3)*	22.1 *(17.6-32.8)*	**0.003**
*Men body mass index (BMI)*	*25.8 (18.4-43.3)*	*22.2 (17.6-32.8)*	***0.002***
Creatinine (μmol/L)	76 *(49–189)*	82 *(45–123)*	0.164
Cockroft clearance (mL/min)	105.7 *(44–257.7)*	90.2 *(67.2-202.9)*	0.244
MDRD (mL/min)	90.9 *(34.2-167.5)*	89.5 *(58.2-144.7)*	0.880
ALT (IU/mL)	62 *(20–383)*	60.5 *(20–1492)*	0.853
AST (IU/mL)	47 *(20–402)*	57 *(26–969)*	0.485
HCV RNA (log IU/mL)	6.1 *(3.59-6.99)*	6.4 *(2.81-7.39)*	0.111
Hemoglobin level (g/dl)	14.5 *(8.2-17.2)*	15.2 *(12.1-18.8)*	**0.036**
*Men hemoglobin level (g/dl)*	*15 (8.3-17.2)*	*15.2 (12.1-18.8)*	*0.239*
HCV genotype n (%)			0.566
1	48 *(76)*	14 *(61)*	
2	2 *(3)*	1 *(4)*	
3	6 *(10)*	4 *(17)*	
4	7 *(11)*	4 *(17)*	
Fibrosis score (Metavir) n (%)			0.780
0 – 2	29 *(46)*	12 *(52)*	
3 – 4	34 *(54)*	11 *(48)*	
HCV treatment status at baseline n (%)			0.434
HCV treatment naive	19 *(30)*	9 *(41)*	
Nonresponders	25 *(40)*	9 *(41)*	
Relapsers	19 *(30)*	4 *(18)*	

ALT, alanine aminotransferase; AST, aspartate aminotransferase; MDRD, Modification of the Diet in Renal Disease. Bold style indicates statistical significance. Quantitative variables are expressed as median and range.

Among HIV-HCV coinfected patients, 20/23 (87%) had a negative HIV RNA viral load at baseline (<50 copies/mL) and 57% had a CD4 count above 500 cells/mm^3^. Most of them received two nucleoside reverse transcriptase inhibitors (NRTIs) (22 patients): tenofovir and emtricitabine for 20 of them (91%) and didanosine associated with stavudine or tenofovir for two. One patient received a cART regimen without NRTI. No patient received abacavir. Nucleoside treatment was associated with a protease inhibitor in 14 patients: seven patients received lopinavir, four darunavir, two atazanavir and one saquinavir. All these patients were also treated with ritonavir as a booster. Eight patients received a non-nucleoside reverse transcriptase inhibitor (NNRTI): four nevirapine, three efavirenz and one etravirine.

Finally, four patients received raltegravir, associated with a protease inhibitor regimen in three of them and with tenofovir and emtricitabine in one. One patient received maraviroc in association with a protease inhibitor, NNRTI and raltegravir.

Medians of AUC_0-4h_ and AUC_0-4h_ normalized to the first RBV dose expressed in mg/kg BW were 1469 μg.h/L and 182 (μg.h/L)/(mg/kg) respectively. According to the threshold of 1755 μg.h/L defined in HCV monoinfected patients, 14/23 (61%) coinfected patients were found to have an AUC_0-4h_ below this value. No significant difference was found for cART type and duration, HIV viral load and CD4 level between the 2 sub-groups of HIV-HCV coinfected patients defined by this threshold (Table 2). All three patients found to have a detectable HIV RNA, had an AUC_0-4h_ below 1755 μg.h/L. With regard to the immune status, 61% of patients had a CD4 level below the retained cutoff of 500/mm^3^. Interestingly, the AUC_0-4h_ was significantly lower for these patients than for those with CD4 level greater than 500/mm^3^ with values of 1270 μg.h/L and 1840 μg.h/L respectively (p = 0.047). For these 2 sub-groups of coinfected patients, normalized AUC were 144 and 195 (μg.h/L)/(mg/kg) but the difference did not reach statistical significance. Table 3 summarizes these results.

**Table 2:** **Particular characteristics of HIV-HCV coinfected patients according to RBV AUC**_**0-4h **_**threshold (n = 23)**

	**All (n = 23)**	**RBV AUC **_**0-4h **_**<1755 μg.h/L (n = 14)**	**RBV AUC **_**0-4h **_**>1755 μg.h/L (n = 9)**	***p value***
HIV RNA <50 copies/mL, n (%)	20 (87)	11 (79)	9 (100)	0.253
HIV RNA copies/mL	50 *(20–9850)*	50 *(20–9850)*	50 *(40–50*)	0.687
CD4 (cells/mm^3^)	543 *(325–1067)*	498 *(325–993)*	543 *(368–1067)*	0.614
CD4%	30 *(10–48)*	29.5 *(10–48)*	32 *(27–45)*	0.174
Duration of HIV treatment (years)	11 *(1–19)*	11 *(1–16)*	10 *(2–19)*	0.825
Antiretroviral therapy, n *(%)*				
Protease inhibitor	14 *(61)*	7 *(50)*	7 *(78)*	
Nucleoside reverse transcriptase inhibitor (NRTI)	21 *(91)*	13 *(93)*	8 *(89)*	
Non-nucleoside reverse transcriptase inhibitor (NNRTI)	8 *(35)*	6 *(43)*	2 *(22)*	
Integrase inhibitor	4 *(17)*	1 *(<1)*	3 *(33)*	
Receptor CCR5 inhibitor	1 *(<1)*	0	1 *(11)*	

Quantitative variables are expressed as median and range.

**Table 3:** **AUC**_**0-4h **_**and normalized AUC**_**0-4h **_**to RBV 600 mg/body weight**

	**HCV (n = 63)**	**HIV-HCV (n = 23)**		***p value***
AUC_0-4h_ (μg.h/L)	2030 (851–7700)	1469 (936–3677)		**0.018**
Patients with AUC_0-4h_ <1755 (%)	32	61		**0.014**
Men AUC_0-4h_ (μg.h/L)	1875 (850–7700)	1414 (1034–3677)		**0.050**
Men with AUC_0-4h_ <1755 (%)	43	65		0.103
Normalized AUC_0-4h_ (μg.h/L)/(mg/kg)	271 (82–1091)	182 (110–425)		**0.001**
Men normalized AUC_0-4h_ (μg.h/L)/(mg/kg)	266 (82–1091)	173 (110–425)		**0.004**
		**CD4 < 500**	**CD4 ≥ 500**	
AUC_0-4h_		1270 (936–2792)	1840 (1280–3677)	**0.047**
Normalized AUC_0-4h_		144 (110–425)	195 (154–349)	0.108

Quantitative variables are expressed as median and range. Bold style indicates statistical significance.

For the reference group of HCV monoinfected patients, median AUC_0-4h_ and normalized AUC_0-4h_ were 2030 μg.h/L and 271 μg.h/L respectively. In comparison to HIV-HCV coinfected patients, these two parameters were significantly higher (p = 0.018 and 0.001, respectively). Sixty-eight percent of monoinfected patients had an AUC_0-4h_ above the threshold of 1755 μg.h/L vs 39% of HIV-HCV coinfected patients (p = 0.014). The median AUC_0-4h_ was slightly higher in naive HCV patients than in relapsers and non-responders (2434 μg.h/L, 2025 μg.h/L and 1873 μg.h/L, respectively, although this difference did not reach statistical significance (p = 0.165).

Different factors potentially associated with RBV pharmacokinetics variability were investigated. Except for body weight, BMI, and hemoglobin level, baseline characteristics did not differ between monoinfected and coinfected patients. Median body weight and BMI were lower in coinfected patients than in monoinfected ones (69 kg vs 75 kg , p = 0.032 and 22.1 vs 25.7, p = 0.003, respectively). Conversely, hemoglobin level was lower in monoinfected patients (median 14.5 g/dl) than in coinfected patients (15.2 g/dl; p = 0.036).

Multivariate logistic regression analysis indicated that presence of HIV coinfection (OR = 3.76, 95% CI [1.21-11.6]; p = 0.022) and male gender (OR = 4.59, 95% CI [1.15-18.3]; p = 0.031) were independently associated with RBV underexposure (Table 4).

**Table 4:** Univariate and multivariate logistic regression analysis to identify factors potentially associated with ribavirin underexposure (defined by an AUC < 1755 μg.h/L)

	**Univariate analysis**		**Multivariate analysis**	
	**OR (95% CI)**	**p**	**OR (95% CI)**	**p**
HIV coinfection (yes *vs* no)	**3.34 (1.24-9.01)**	**0.017**	**3.76 (1.21-11.6)**	**0.022**
Gender (male *vs* female)	**7.00 (1.89-25.9)**	**0.004**	**4.59 (1.15-18.3)**	**0.031**
Weight	1.03 (0.99-1.06)	0.067	1.03 (0.99-1.08)	0.142
Age	0.97 (0.93-1.01)	0.160	-	-
MDRD	1.00 (0.98-1.02)	0.803	-	-
Cockroft	1.01 (0.99-1.02)	0.145	1.00 (0.98-1.02)	0.927

Bold style indicates statistical significance.

## Discussion

Using the AUC_0-4h_ method, our results clearly showed a plasma underexposure in HIV-HCV coinfected patients after a single dose intake of RBV in comparison with monoinfected patients. The pharmacokinetics of RBV is less documented in HIV-HCV coinfected patients than in monoinfected ones but some studies showed that the achievement of RBV cutoffs was a predictive factor of SVR independent of HIV coinfection [20,21]. The heterogeneity of our cohort of HIV-HCV coinfected patients was representative of a treated HIV population in a hospital environment (57% with CD4 > 500 cells/mm^3^; 87% with undetectable HIV viral load under HAART; patients mainly under 2 NRTIs + 1 boosted protease inhibitor) [22]. The choice of AUC_0-4h_ was made in accordance with the study of Loustaud-Ratti *et al.* for HCV monoinfected patients [17]. Indeed, the authors showed that the abbreviated AUC_0-4h_ was highly correlated to the full AUC_0-12h_ because it described the main inter-individual variability of RBV pharmacokinetic parameters (i.e. absorption and distribution phases). Interestingly in our study, more than 60% of coinfected patients had an AUC_0-4h_ below the threshold of 1755 μg.h/L defined as associated with SVR in HCV monoinfected patients [17]. In comparison, only 32% of monoinfected patients of our study had an AUC_0-4h_ below this value. Demographic or biological parameters were collected in order to detect confounding factors that could affect differences in early RBV exposure between both populations. Some differences were found. The first of them was a body weight significantly lower for coinfected patients. As the initial RBV dose was the same (600 mg) for all patients, irrespectively of this parameter, early exposure was normalized for RBV dose expressed as mg/kg BW. Normalized AUC_0-4h_ remained significantly different between both groups. In addition, hemoglobin level was higher in coinfected than in monoinfected patients. It is well known that RBV is highly concentrated in red blood cells [23], but this parameter was not expected to influence AUC_0-4h_ which was determined after a single dose of RBV. Among other parameters known to have an influence on RBV exposure is the renal function, but neither median baseline serum creatinine, nor estimated glomerular filtration rate (GFR) calculated by Cockroft-Gault equation or MDRD formula, differed significantly between both groups (Table 1). We have determined early RBV exposure regardless of HCV genotype as no difference was observed in the genotype distribution in each of the two populations of this study and because we wanted to evaluate early bioavailability of RBV independently of the subsequent treatment response. In the study by Loustaud-Ratti *et al.* the population was exclusively constituted of monoinfected HCV genotype 1 patients. This is the main difference with our study in which genotypes 1 to 4 were present but with a majority of genotype 1. Indeed, except for this parameter and for the distribution of fibrosis score, baseline characteristics were similar in both studies. However, 32% of our HCV monoinfected patients had an AUC_0-4h_ below the 1755 μg.h/L threshold compared with 58% in this historical study. This difference could be explained by the presence of patients infected by HCV genotype 2, 3 or 4 because only two of these 15 patients had an AUC_0-4h_ below this threshold. Finally, we found a strong gender ratio in favor of male patients in our coinfected population. In order to estimate if gender could affect RBV exposure, we compared parameters which were statistically different in the whole population. For body weight the significant difference was even more important and as expected, this parameter was lower in coinfected patients (Table 1). Hence the interest of the AUC normalized to the first RBV dose deletes the weight effect. Conversely, differences in hemoglobin level were no longer significant due to the higher proportion of women in the monoinfected group. In spite of the reduced number of patients, the difference of AUC_0-4h_ or normalized AUC_0-4h_ between monoinfected and coinfected male patients was statistically significant, and the proportion of AUC under the 1755 μg.h/L threshold remained high. No other differences in demographic or biological characteristics were found between the two populations of this study.

Given that there was a strong gender ratio in favor of male patients and in order to see if gender could affect our results, we compared AUC_0-4h_ between monoinfected and coinfected male patients. It is noteworthy that 43% of monoinfected and 65% of coinfected male patients had AUC_0-4h_ below the 1755 μg.h/L threshold, although this difference did not reach statistical significance (p = 0.103). As expected, among HCV monoinfected patients, the AUC_0-4h_ was slightly higher in naive patients than in relapsers and non-responders to a previous treatment although this difference did not reach statistical significance probably because of a limited number of patients in each subgroup.

Unexpectedly, RBV plasma underexposure seems to be associated with the immunological level of HIV patients. RBV exposure was indeed better if CD4 cell count was greater than 500/mm^3^. In the RIBAVIC study, using PEG-IFN/RBV therapy in HIV co-infected patients, a trend for a lower SVR was noted in patients with a CD4 cell count under 500 versus patients upper this threshold (21% vs 33%, respectively; p = 0.051) [24]. Then, in the PRESCO trial, a higher dosing of ribavirin (weight-based dosing with 1000 to 1200 mg) was associated with better SVR rates but higher risk of anemia [25]. In the present study, when the immunorestoration under cART was considered complete, i.e. with a CD4 cell count > 500/mm^3^, RBV exposure was similar in this HIV sub-group in comparison with HCV monoinfected population.

Food intake is known to potentially impact RBV bioavailability. Both the AUC_tf_ (from time zero to the time of the final quantifiable sample) and C_max_ of a single oral dose of ribavirin were indeed shown to be increased by co-administration of a high fat meal [26]. However, in the present study all patients received RBV with light breakfast or snack which suggests no bias between mono- and coinfected patients.

## Conclusion

In conclusion, HIV-HCV coinfected patients present a lower RBV exposure which could at least in part explain the lower response rate of these patients to HCV treatment. This hypothesis should be further investigated on a larger number of coinfected patients focusing on the immune status of these patients. In the past, recommendations were to use low dose of RBV in HIV patients in order to avoid toxicity associated with antiretroviral drugs, such as zidovudine, stavudine or didanosine [27,28]. However, these drugs are progressively neglected to the benefit of less cytotoxic new antiretroviral drugs. So, higher doses of RBV can be used to improve SVR in coinfected patients, as suggested in several studies [7,29]. Measurement of early RBV exposure by determination of AUC_0-4h_ possibly followed by RBV dose adjustment could help in optimizing antiviral therapy in HIV-HCV coinfected patients, particularly in the more immunosuppressed ones because RBV underexposure was noted in this specific sub-group.

## Abbreviations

AUC: Area under the curve; BMI: Body mass index; cART: Effective combination antiretroviral therapy; GFR: Glomerular filtration rate; HAART: Highly active antiretroviral therapy; HCV: Hepatitis C virus; HIV: Human immudeficiency virus; MDRD: Modification of the diet in renal disease; RBV: Ribavirin; SVR: Sustained virological response.

## Competing interests

Fabien Zoulim received consulting/speaker fees from Gilead Science, Bristol Myers Squibb, and Roche. François Bailly received speaker fees from Janssen. The other authors have no conflict of interests to declare. No financial support.

## Authors’ contributions

FB, PP, MM, FP, FZ, MCG participated in the conception and design of the study. GH, FB, EP, PP, PM, MM, FP, PC, JML participated in data collection. EP and PP performed the statistical analysis. GH, FB, EP, PP, PM and MCG helped to draft the manuscript. All authors read and approved the final version of the manuscript.

## Pre-publication history

The pre-publication history for this paper can be accessed here:

http://www.biomedcentral.com/1471-2334/14/150/prepub
